# The Alpha-Tocopherol Form of Vitamin E Boosts Elastase Activity of Human PMNs and Their Ability to Kill *Streptococcus pneumoniae*

**DOI:** 10.3389/fcimb.2017.00161

**Published:** 2017-05-03

**Authors:** Elsa N. Bou Ghanem, James N. Lee, Basma H. Joma, Simin N. Meydani, John M. Leong, Alexander Panda

**Affiliations:** ^1^Department of Molecular Biology and Microbiology at Tufts, University School of MedicineBoston, MA, USA; ^2^Program in Immunology, Sackler School of Graduate Biomedical Sciences, Tufts UniversityBoston, MA, USA; ^3^Jean Mayer USDA Human Nutrition Research Center on Aging at Tufts UniversityBoston MA, USA

**Keywords:** vitamin E, aging, neutrophils, *S. pneumoniae*, infection, inflammation, serine proteases

## Abstract

Despite the availability of vaccines, *Streptococcus pneumoniae* remains a leading cause of life-threatening infections, such as pneumonia, bacteremia and meningitis. Polymorphonuclear leukocytes (PMNs) are a key determinant of disease course, because optimal host defense requires an initial robust pulmonary PMN response to control bacterial numbers followed by modulation of this response later in infection. The elderly, who manifest a general decline in immune function and higher basal levels of inflammation, are at increased risk of developing pneumococcal pneumonia. Using an aged mouse infection model, we previously showed that oral supplementation with the alpha-tocopherol form of vitamin E (α-Toc) decreases pulmonary inflammation, in part by modulating neutrophil migration across lung epithelium into alveolar spaces, and reverses the age-associated decline in resistance to pneumococcal pneumonia. The objective of this study was to test the effect of α-Toc on the ability of neutrophils isolated from young (22–35 years) or elderly (65–69 years) individuals to migrate across epithelial cell monolayers in response to *S. pneumoniae* and to kill complement-opsonized pneumococci. We found that basal levels of pneumococcal-induced transepithelial migration by PMNs from young or elderly donors were indistinguishable, suggesting that the age-associated exacerbation of pulmonary inflammation is not due to intrinsic properties of PMNs of elderly individuals but rather may reflect the inflammatory milieu of the aged lung. Consistent with its anti-inflammatory activity, α-Toc treatment diminished PMN migration regardless of donor age. Unexpectedly, unlike previous studies showing poor killing of antibody-opsonized bacteria, we found that PMNs of elderly donors were more efficient at killing complement-opsonized bacteria *ex vivo* than their younger counterparts. We also found that the heightened antimicrobial activity in PMNs from older donors correlated with increased activity of neutrophil elastase, a serine protease that is required to kill pneumococci. Notably, incubation with α-Toc increased PMN elastase activity from young donors and boosted their ability to kill complement-opsonized pneumococci. These findings demonstrate that α-Toc is a potent modulator of PMN responses and is a potential nutritional intervention to combat pneumococcal infection.

## Introduction

Despite the availability of vaccines and antibiotics, *Streptococcus pneumoniae* (pneumococcus) still causes invasive pneumococcal diseases, including pneumonia, meningitis and bacteremia (Chong and Street, 2008), particularly in individuals >65 years old (Plosker, 2015). In the US, the elderly account for 60% of hospitalizations due to this infection, resulting in an estimated direct cost of $2.5 billion annually (Wroe et al., 2012). A cell type that plays an important role in host defense against *S. pneumoniae* infections is the neutrophil (polymorphonuclear leukocyte, or PMN) (Garvy and Harmsen, 1996; Bou Ghanem et al., 2015). Studies from our laboratory and others have shown that PMNs are required to control bacterial burden early in the infectious process (Garvy and Harmsen, 1996; Hahn et al., 2011; Bou Ghanem et al., 2015), but poorly controlled PMN influx into the lung airways can lead to tissue destruction and promote the spread of infection (Bhowmick et al., 2013). In fact, we found that immunodepletion of PMNs 18 h after infection promoted host survival in a murine model of pneumococcal pneumonia (Bou Ghanem et al., 2015). These findings suggest that host survival necessitates an immediate PMN response followed by resolution later in the course of *S. pneumoniae* lung infection.

We previously showed that compared to young mice, aged mice exhibited greater PMN recruitment into the lungs following *S. pneumoniae* challenge (Bou Ghanem et al., 2014). In humans, baseline PMN numbers are elevated in the lungs of healthy elderly volunteers (Pignatti et al., 2011), and elderly *S. pneumoniae-*infected patients exhibit higher infiltration of neutrophils in lung tissue compared to younger patients (Menter et al., 2014). The potential role of increased bacterial loads, intrinsic PMN behavior, or the signaling by the pulmonary environment in exacerbating the influx of PMNs in aged individuals is unclear. Inflammatory cytokines, including the potent PMN chemoatractant IL-8, are elevated (Meyer et al., 1996, 1998; Krone et al., 2014) in the lungs of the elderly. However, the chemotactic response of PMNs isolated from elderly donors to sputum from *S. pneumoniae* patients is blunted compared to PMNs from younger donors (Sapey et al., 2014).

Efficient killing of *S. pneumoniae* by human PMNs requires phagocytosis (Standish and Weiser, 2009). Both complement and antibodies can mediate opsonophagocytic uptake and killing of *S. pneumoniae* (Esposito et al., 1990). Pneumococci that are opsonized with the combination of rabbit complement and antibodies from the sera of an immunized young donor are killed less efficiently by PMNs from elderly donors than by their young counterparts, suggesting that antibody and/or complement-mediated opsonophagocytic killing by PMNs diminishes with age (Simell et al., 2011). An age-related decline in antibody-mediated killing (Fulop et al., 1985) may be related to a decline in levels and opsonic capacity of antibodies against pneumococci (Park and Nahm, 2011; Simell et al., 2011) as well as FcRIII (CD16) expression on PMNs (Butcher et al., 2001). In the absence of an antibody response, individuals rely on complement for opsonization (Standish and Weiser, 2009; Dalia et al., 2010), and serum complement activity and PMN complement receptors expression remain unchanged or increase upon aging (Bellavia et al., 1999; Simell et al., 2011). However, the effect of aging on complement-mediated opsonophagocytic killing remains unclear.

Pneumococcal killing by PMNs is independent of oxidative burst (Marriott et al., 2008; Standish and Weiser, 2009) but dependent on serine proteases cathepsin G (CG), neutrophil elastase (NE) and proteinase 3 (Standish and Weiser, 2009; Hahn et al., 2011). These degradative enzymes are typically prepackaged into azurophilic granules during PMN development in the bone marrow (Gullberg et al., 1997; Pham, 2006; Cowland and Borregaard, 2016). They are thought to be released upon fusion of PMN granules with the phagolysosome after ingestion of microbes (Pham, 2006) but can also be released into in the extracellular space to kill microbes independent of phagocytosis (Pham, 2006; Standish and Weiser, 2009). Enzymatic inhibition of these proteases diminishes intracellular killing of pneumococci by PMNs (Standish and Weiser, 2009) and mice deficient for neutrophil elastase and/or cathepsin G demonstrate enhanced susceptibility to pneumococcal lung infection (Hahn et al., 2011). Interestingly, sera and bronchoalveolar lavage from donors >65 years old exhibit an increase in neutrophil elastase levels and activity (Varga et al., 1992; Meyer et al., 1998; Paczek et al., 2009).

Elderly are at an increased risk of inadequate intake of vitamin E (Panemangalore and Lee, 1992; Ryan et al., 1992), an anti-oxidant with potent immunoregulatory functions (Wu and Meydani, 2008). Several naturally occurring forms of vitamin E exist (Pae et al., 2012), and dietary supplementation of alpha-tocopherol, the most bioavailable form, was shown to enhance adaptive immune responses (Meydani et al., 1997; Adolfsson et al., 2001; Marko et al., 2007). Using a murine model, we previously showed that supplementation with alpha-tocopherol vitamin E (referred to here as α-Toc) reversed the age-associated susceptibility to pneumococcal infection at least in part by modulating pulmonary recruitment of PMNs, resulting in a 1,000-fold lower bacterial lung burden and control of infection (Bou Ghanem et al., 2014). α-Toc inhibited the migration of PMNs isolated from young donors across cultured epithelial cell monolayers in response to *S. pneumoniae* infection, altering the expression of multiple PMN and epithelial cell adhesion molecules involved in migration (Bou Ghanem et al., 2014). The effect of vitamin E on the migration and antibacterial responses of PMNs, and whether its efficacy in modulating these responses differs between young vs. elderly donors, remain unexplored.

## Materials and methods

### Donors

All procedures in this study were approved by Tufts Medical Center Human Investigation Review Board (IRB). Young (22–35 years) and elderly (65–69 years) healthy human volunteers were recruited through the HNRCA Volunteer Services Department and the Department of Molecular Biology and Microbiology, Tufts University School of Medicine. All enrolled subjects signed IRB approved consent forms. Individuals that were pregnant, taking medication or reporting symptoms indicative of infection within the prior 2 weeks were excluded from the study. Blood was drawn between 8 am and 10 am and study subjects were asked not to have any food intake after midnight on the day of blood draw.

### Bacteria

*Streptococcus pneumoniae* TIGR4 strain (serotype 4), were grown at 37°C with 5% CO_2_ in Todd-Hewitt broth (BD Biosciences) supplemented with 0.5% yeast extract and Oxyrase (Oxyrase) till mid-exponential phase. Aliquots were then frozen at −80°C in the growth media with 25% (v/v) glycerol. Before use, bacterial aliquots were thawed on ice, washed once and diluted in PBS to the required concentrations. Bacterial titers were confirmed by plating on Tryptic Soy Agar plates supplemented with 5% sheep blood agar (Northeast Laboratory Services).

### Isolation of human PMNs

Whole blood was drawn using acid citrate/dextrose as an anti-coagulant. PMNs were then purified using a 2% gelatin sedimentation as previously described (Bou Ghanem et al., 2014). This method allows for isolation of active PMNs with ~90% purity.

### *In vitro* vitamin E treatment of PMNs

A vitamin E stock solution (ADM) of 30 mg/ml of d-α-tocopherol in ethanol was prepared. To increase cellular uptake (Marko et al., 2007) the stock solution was diluted in FBS to a final concentration of 2 mg/ml and incubated for 30 min at 37°C with gentle vortexing at 10 min intervals. PMNs (2 × 10^7^ cells/ml) were incubated with the α-tocopherol form of vitamin E (α-Toc) at a final concentration of 25 μg/ml or with 0.06% ethanol as vehicle control in HBSS lacking calcium and magnesium for 1 h, washed and then added to the Transwells for the migration assay or used in the opsonophagocytic assay (detailed below).

### PMN migration assay across lung epithelial cells

Transmigration assay was performed as previously described (Bou Ghanem et al., 2014). Briefly, Human pulmonary mucoepidermoid carcinoma-derived NCI-H292 (H292) (ATCC) cells were seeded on inverted Transwell filters collagen-coated Transwell filters (0.33-cm^2^, Corning Life Sciences) and then allowed to grow and polarize for 1 week in RPMI 1640 medium (ATCC) with 2 mM L-glutamine, 10% FBS (Invitrogen) and 100 U penicillin/streptomycin. On the day of the migration assay, the epithelial monolayers were washed out of the antibiotic-containing media and equilibrated in HBSS for 30 min. The apical side of monolayers was infected with *S. pneumoniae* at a multiplicity of infection (MOI) of 20 for 2.5 h. Uninfected wells were treated with HBSS. The monolayers were washed and placed into 24-well plates. 600 μl of HBSS was added to the lower (apical) chamber and 100 μl of PMNs (1 × 10^6^) was added to the top (basolateral) chamber. PMNs that transmigrated into the apical chamber were collected and lysed in 10% Triton-X 100. Migrated neutrophils were quantitated using myeloperoxidase ELISA following a well-established assay (McCormick et al., 1995) where serial dilutions of known numbers of neutrophils were used to establish a standard curve. For each donor the average migration from triplicate wells per condition was assessed and plotted.

### Opsonophagocytic killing assay

The ability of human PMNs to kill pneumococci was assessed *ex vivo* as described previously (Dalia et al., 2010) with some modifications. Briefly, 5 × 10^5^ PMNs were incubated with 1 × 10^3^ bacteria grown to mid log phase and pre-opsonized with 10 μl rabbit complement (pel-freez) in 200 μl reactions in Hank's buffer/0.1% gelatin. The reactions were incubated rotating for 45 min at 37°C. Percent killing in comparison to incubations with no PMNs was determined by plating serial dilutions on blood agar plates. For each donor six total replicates were performed and *n* = 3 were plated to calculate efficiency of killing and the other three used for quantification of serine proteases (described below). To measure if bacterial uptake by PMNs decreased with age, we followed a previously published assay where bacteria are pre-labeled with a Fluorescein isothiocyanate (FITC) dye and phagocytosis is assessed by flow cytometry following addition of trypan blue that quenches any extracellular signal allowing detection of internalized bacteria (Dalia et al., 2010). We saw that age had no effect on the percentage of PMNs that were FITC-positive, however, although FITC-labeling of bacteria did not have any significant effect on bacterial viability directly, it did interfere with the ability of PMNs to kill pneumococci (data not shown).

### Cathepsin G and neutrophil elastase assays

For each donor, pooled PMN pellets from three opsonophagocytic reactions (1.5 × 10^6^ PMNs) were lysed with 300 μl of the Cathepsin G Activity Assay Kit (abcam) lysis buffer. PMN elastase levels (abcam PMN Elastase Human ELISA Kit) and activity (abcam PMN Elastase Fluorometric Activity Assay Kit) as well as Cathepsin G levels (Aviva Systems Biology CTSG Human ELISA Kit) and activity (abcam Cathepsin G Activity Assay Kit) in the cell pellets were then determined following manufacturer's instructions.

### Statistics

All statistical analysis was performed using Prism5 for Macintosh (Graph Pad). D'Agostino & Pearson omnibus normality test was used to determine if the data were normally distributed. Paired student *t*-test, unpaired student *t*-test or Mann-Whitney test were used for comparison between groups as indicated. Pearson or Spearman tests were used to determine correlation. One sample *t*-test was used to measure whether α-Toc -indices were different from 1.0. All comparisons with *p* < 0.05 were considered significantly different. Individual points representing data from a single donor were plotted. Lines and error bars represent the mean values ± SEM.

## Results

### Aging has no impact on PMN transepithelial migration in response to pneumococcal infection

**Figure 1:**
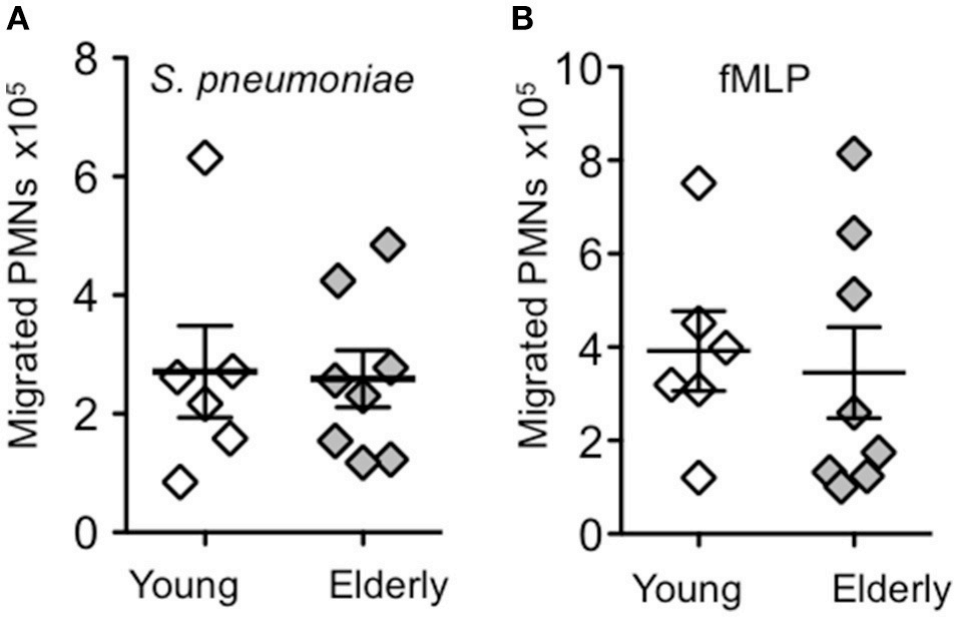
**Aging has no impact on PMN transepithelial migration in response to pneumococcal infection**. The number of PMNs that migrated from the basolateral to the apical side of polarized H292 epithelial cells in response to **(A)** pneumococcal infection or **(B)** fMLP were measured by MPO ELISA (see Materials and Methods). Data from 6 young donors and 8 elderly donors are shown. For each donor the average number of migrated PMNs from three technical replicates is shown. There was no significant difference in migration between age groups by unpaired Student's *t*-test.

Prior studies demonstrated that aging was associated with an increase in PMN recruitment in to the lungs following *S. pneumoniae* infection (Bou Ghanem et al., 2014; Menter et al., 2014). To test whether PMNs from elderly individuals possess an enhanced intrinsic ability to migrate in response to pneumococcal infection, we measured PMN migration across lung epithelial cells *in vitro*. Polarized monolayers of H292 human epithelial cells on Transwell filters were mock-infected or apically infected with *S. pneumoniae* TIGR4 at an MOI of 20. PMNs from young (22–35) or elderly (65–69) healthy human volunteers were then added to the basolateral side and cumulative PMN migration was measured after 120 min. No detectable migration was observed in the absence of infection in either group (data not shown). As previously observed (Bou Ghanem et al., 2014), *S. pneumoniae* infection elicited robust trans-epithelial movement of PMNs from all young donors, with some variation in the ability of PMNs from different donors to migrate (2.7 ± 1.9 × 10^5^; Figure 1A). Transepithelial migration by PMNs isolated from elderly donors (2.6 ± 1.4 × 10^5^; Figure 1A) was indistinguishable from that of young controls. PMN migration across the epithelium in response to the chemoattractant peptide N-formyl-methionyl-leucyl-phenylalanine (fMLP) was also indistinguishable between the age groups (Figure 1B).

### α-Toc reduces pneumococcus-induced transepithelial migration of PMNs from both young and elderly donors

**Figure 2:**
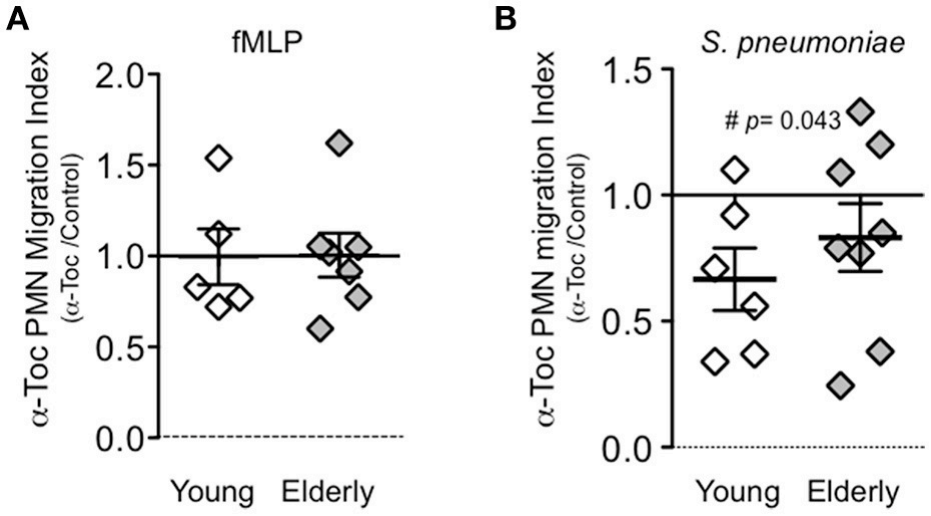
**α-Toc reduces pnemococcus-induced transepithelial migration of PMNs from both young and elderly donors**. PMNs were pre-treated with α-Toc or vehicle control and allowed to migrate across polarized lung epithelial cells in response to **(A)** fMLP or pneumococcal infection **(B)**. The average number of migrated PMNs for each donor was calculated from three technical replicates per condition. For each donor α-Toc PMN migration index (y-axis) was calculated by dividing the average number of α-Toc pre-treated transmigrated PMNs by the average number of vehicle control treated transmigrated PMNs. Data from 6 young donors and 8 elderly donors are shown. Unpaired Student's *t*-test revealed no significant difference in the effect of α-Toc on PMN migration between age groups. One sample *t*-test revealed α-Toc migration index was significantly different from 1 only in the young donors (*p* < 0.05 denoted by #).

We previously found that vitamin E mitigates the harmful inflammatory response during pneumococcal infection of aged mice partially due to its direct effect on PMN migration (Bou Ghanem et al., 2014). To test the effect of vitamin E on the transmigration response of PMNs from elderly individuals, PMNs were pretreated with 25 μg/ml of α-tocopherol form of vitamin E (α-Toc), a concentration that was previously found to blunt migration of PMNs from young donors and is within the range of levels measured in plasma of humans taking a daily supplement of 200 International Units of vitamin E (Meydani et al., 1997). This concentration of α-Toc had no effect on either PMN or bacterial viability (data not shown) (Bou Ghanem et al., 2014). α-Toc treatment significantly reduced PMN migration in 5 out of 8 elderly donors and 4 out of 6 young donors tested (*p* < 0.05 by paired *t*-test). To more thoroughly analyze the magnitude of the effect of α-Toc, for PMNs from each individual tested for transepithelial migration in the presence or absence of α-Toc, we calculated a “α-Toc PMN migration index,” which is the ratio of migration in the presence of α-Toc to migration in its absence (Figure 2). As previously observed, α-Toc treatment had no effect on fMLP-induced migration of PMNs from young donors (Bou Ghanem et al., 2014), and here we found that α-Toc also had no effect on migration of PMNs from aged donors, with α-Toc migration indices of close to 1.0 (Figure 2A). In contrast, PMN migration in response to pneumococcal infection from young and elderly donors was diminished by α-Toc treatment (Figure 2B), with average α-Toc migration indices of 0.66 and 0.83, respectively. Although the α-Toc migration indices were not significantly different between the age groups (*p* = 0.39 as measured by Student's *t*-test), α-Toc treatment significantly reduced average migration in PMNs isolated from young (*p* = 0.043), but not elderly (*p* = 0.249) donors as measured by one sample *t*-test. Finally, although α-Toc treatment blunted PMN transepithelial migration in response to pneumococcal infection in the majority of individuals, the migration of PMN of some individuals was either not affected or slightly increased by α-Toc (Figure 2B), consistent with previous findings that the effect of vitamin E can vary between individuals (Belisle et al., 2010).

### Aging is associated with more efficient killing of complement-opsonized pneumococci by PMNs

**Figure 3:**
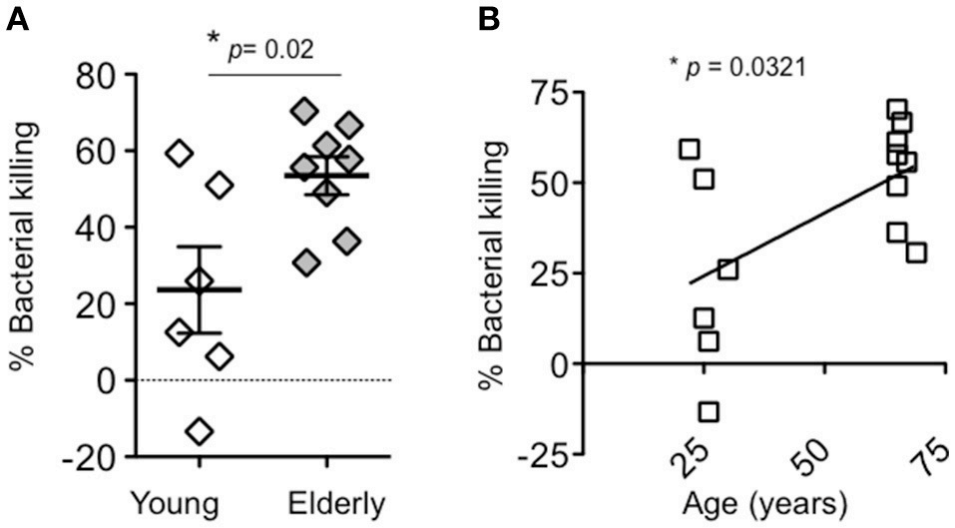
**Aging is associated with more efficient killing of complement-opsonized pneumococci by PMNs. (A)** The percentage of complement-opsonized *S. pneumoniae* TIGR4 killed upon a 45-min incubation with PMNs isolated from young and elderly donors was determined by comparing surviving CFU to a no PMN control. Shown are the averages of triplicate reactions from 6 young donors and 8 elderly donors within the same experiment. Significance was determined by unpaired student's *t*-test. **(B)** Pearson correlation analysis revealed a significant (*p* < 0.05) correlation between PMN bacterial killing and age, denoted by “^*^.”

The absence of age-specific transepithelial migration responses left open the question whether intrinsic PMN anti-pneumococcal function declines with age. Throughout their lifetimes, the majority of individuals are intermittently nasopharyngeally colonized by *S. pneumoniae* strains (Simell et al., 2012), a process that can generate a humoral immune response (Ferreira et al., 2013). Therefore, rather than opsonizing bacteria with (potentially antibody-containing) donor sera, which could confound our interpretation, we opsonized bacteria with purified complement prior to incubation with PMNs isolated from young and elderly donors. Surprisingly, PMNs isolated from elderly donors killed complement-opsonized bacteria significantly more efficiently than their younger counterparts (Figure 3A), and analysis of bacteriocidal activity after stratification by age revealed a significant positive correlation between bacterial killing and age (Figure 3B).

### Aging is associated with increased neutrophil elastase activity

**Figure 4:**
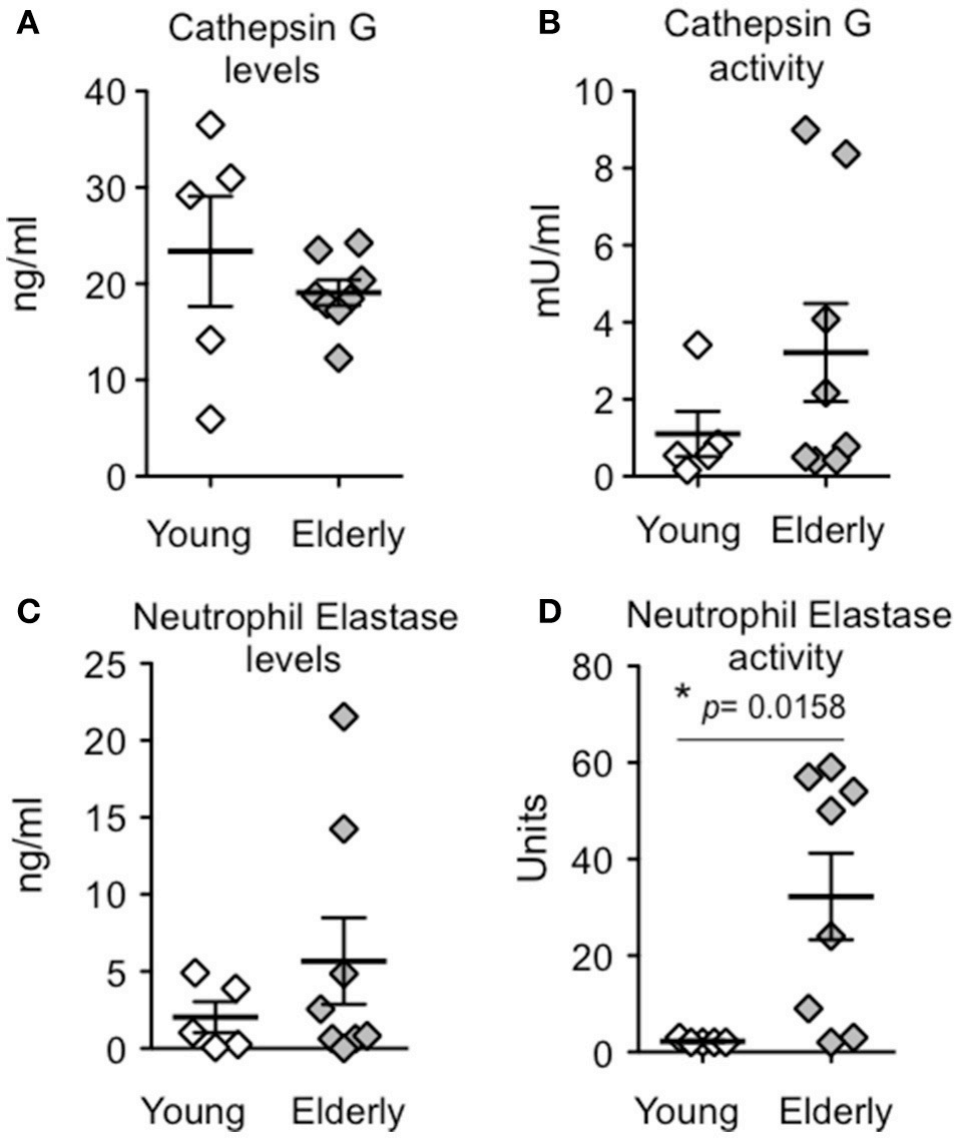
**Aging is associated with increased PMN neutrophil elastase activity**. PMNs from young or elderly donors were incubated for 45 min with complement-opsonized *S. pneumoniae* TIGR4. The level **(A,C)** and activity **(B,D)** of cathepsin G or neutrophil elastase in PMN lysates were determined (see Materials and Methods). Data from 5 young and 8 elderly donors are shown. Significant differences were determined by a Mann-Whitney test and denoted by “^*^.”

Killing of engulfed pneumococci by human PMNs was previously shown to be dependent on serine proteases, such as cathepsin G (CG) and neutrophil elastase (NE) (Standish and Weiser, 2009), so we compared the amounts and activities of these proteases in young or aged *S. pneumoniae*-infected PMNs (see Materials and Methods). First, irrespective of donor age, we detected very low amounts and activities of both cathepsin G and neutrophil elastase in the supernatants following pneumococcal infection of PMNs (data not shown), suggesting that *S. pneumoniae* infection does not trigger large scale exocytosis of serine protease-containing granules in our assay. Second, ELISAs of cell pellets revealed no significant age-associated difference in cathepsin G amounts (Figure 4A); activity measurements showed that PMNs isolated from elderly donors had on average 3-fold higher cathepsin G activity compared to young PMNs, but this difference did not reach statistical significance (*p* = 0.16; Figure 4B). Neutrophil elastase levels did not significantly differ between groups (Figure 4C), but PMNs from elderly donors displayed a significant (*p* = 0.0158), 15-fold higher level of neutrophil elastase activity (Figure 4D) compared to young PMNs. Finally, *F*-test of variances indicated significantly (*p* < 0.05) more individual-to-individual variation in activity levels of both cathepsin G and neutrophil elastase in PMNs from elderly donors compared to young controls. These data suggest that, in the majority of subjects, aging is accompanied by increased activity of serine proteases, which could contribute to the heightened intrinsic anti-microbial activity of PMNs from elderly donors.

### α-Toc boosts serine protease activity and bacterial killing by PMNs from young donors

**Figure 5:**
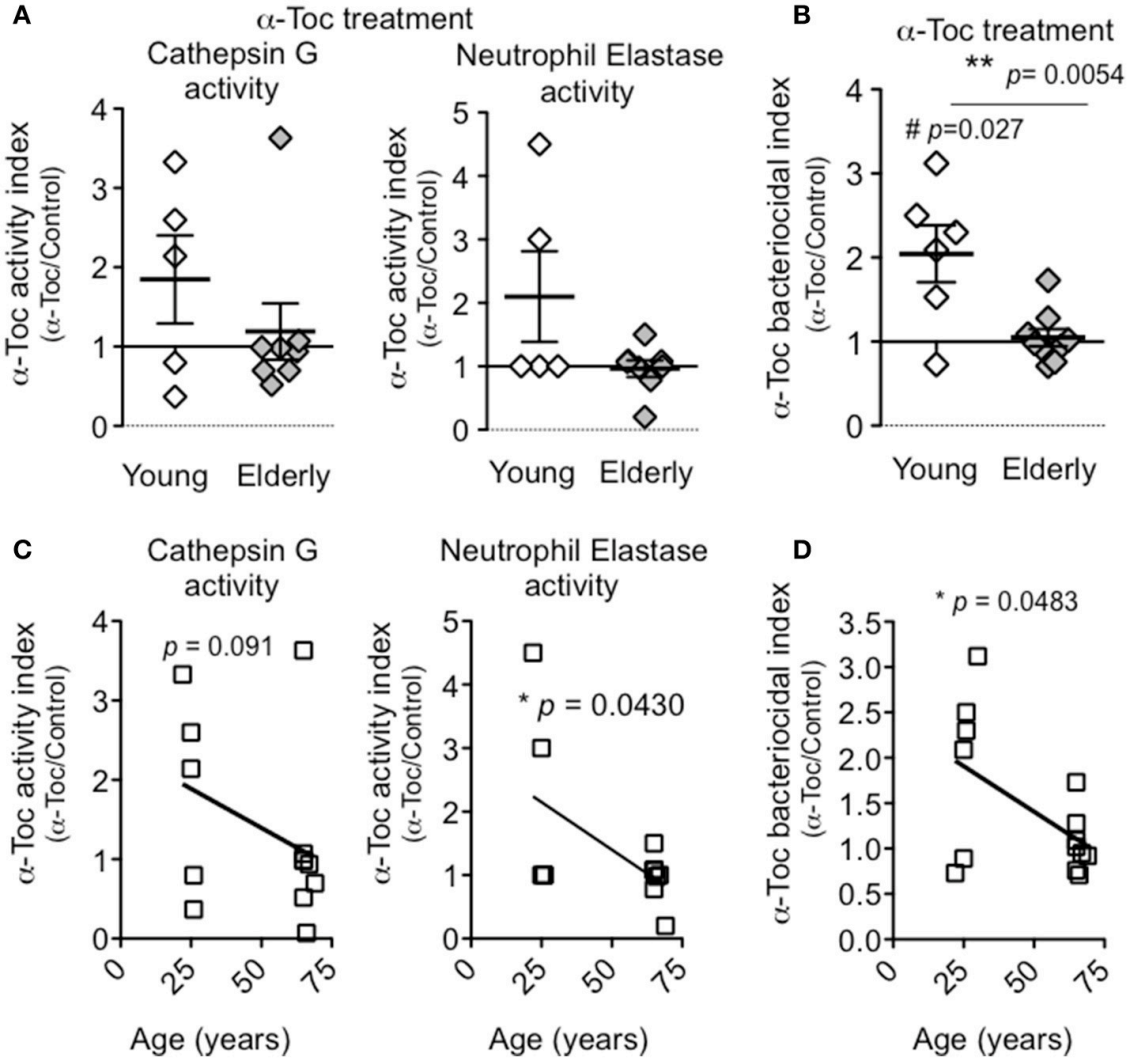
**α-Toc boosts bacterial killing by PMNs from young donors**. PMNs were pre-treated with α-Toc or vehicle control and then incubated for 45 min with complement-opsonized *S. pneumoniae* TIGR4. **(A)** The activity of cathepsin G (left panel) and neutrophil elastase (right panel) in lysates of pelleted PMNs were determined (see Materials and Methods). Shown are the α-Toc activity indices, i.e. the ratio of activities of α-Toc-treated to control-treated PMNs, for all donors. Significant differences were determined by Mann Whitney test. **(B)** The average percent bacterial killing compared to a no PMN control was calculated from triplicate wells per condition (see Materials and Methods). Shown are the α-Toc bacteriocidal indices, i.e. the ratio of killing by α-Toc-treated to control-treated PMNs, for all donors. Data from 5 young donors and 8 elderly donors are shown. Significant differences were determined by unpaired Student's *t*-test (*p* < 0.05 denoted by ^**^) and one sample *t*-test revealed α-Toc bacteriocidal index was significantly different from 1 only in the young donors (*p* < 0.05 denoted by #). **(C)** Spearman correlation analysis between α-Toc activity index and age and **(D)** Pearson correlation analysis between α-Toc bacteriocidal index and age revealed significant correlations denoted by “^*^.”

We next tested the effect of α-Toc on the serine protease and bacteriocidal activities of PMNs isolated from young and elderly donors following exposure to *S. pneumoniae ex vivo*. For both activities, we determined the respective α-Toc indices, i.e., the ratio of activity in the presence of α-Toc to the activity in its absence. The baseline activity of cathepsin G and neutrophil elastase was elevated in PMNs from aged donors compared to young donors (Figure 4), and the α-Toc treatment did not increase these activities in PMNs from aged donors any further, resulting in α-Toc activity index of 1.1 and 0.96 for cathepsin G and neutrophil elastase, respectively (Figure 5A). The α-Toc bacteriocidal index of PMNs from elderly donors also was 1.0, indicating that α-Toc had no effect on the ability of these PMNs to kill pneumococci in our assay (Figure 5B). In contrast, the average α-Toc activity indices for cathepsin G and neutrophil elastase from PMNs from young donors were 1.85 and 2.1, respectively. These α-Toc activity indices were not significantly higher than 1.0 or than the activity indices of PMNs from elderly donors. The average α-Toc bacteriocidal index in young donors, at 2.0, was elevated and was both significantly higher than 1.0 (*p* = 0.027) and significantly higher than that of elderly donors (*p* = 0.0054; Figure 5B). In fact, while α-Toc treatment significantly (*p* < 0.05) boosted PMN killing in only 1 out of the 8 elderly donors by paired *t*-test, PMNs from 4 out of 6 young donors tested showed significantly increased bactericidal activity upon exposure to α-Toc. Furthermore, statistical analyses upon numeric stratification by age revealed that the ability of α-Toc to boost neutrophil elastase activity and bacterial killing both negatively correlated with age (Figures 5C,D), suggesting that the effect of α-Toc on PMN function is age-dependent. These findings suggest that α-Toc boosts the ability of PMNs from young donors to kill *S. pneumoniae*, in part by increasing the activity of anti-microbial serine proteases, in particular neutrophil elastase.

## Discussion

It is unclear to what extent age-driven changes in intrinsic PMN function, as opposed to PMN-independent changes in the pulmonary environment, contribute to the exacerbated inflammatory responses during pneumococcal infection. Compared to young controls, the aged lung is associated with elevated levels of proinflammatory cytokines and epithelial surface adhesion receptors both at baseline and upon pneumococcal infection (Shivshankar et al., 1990; Meyer et al., 1996, 1998; Hinojosa et al., 2009). Some studies suggest age-related dysregulation of PMN behavior, because chemotaxis of PMNs toward fMLP, IL-8 or sputum from patients with pneumococcal pneumonia becomes non-linear and slowed upon aging (MacGregor and Shalit, 1990; Sapey et al., 2014). In the current study, using a relatively limited number of human donors, we found no difference in the ability of PMNs from young and old donors to migrate across monolayers of epithelial cells in response to fMLP or *S. pneumoniae* infection. This suggests that the increase of pulmonary inflammation associated with aging is not due to intrinsic properties of PMNs of elderly individuals, but rather may reflect the inflammatory environment of the aged lung. Given that migration across cultured respiratory epithelium in this experimental system requires 12-lipoxygenase, an enzyme critical for the production of the eicosanoid PMN chemoattractant hepoxilin A3 (Bhowmick et al., 2013), these studies suggest that the intrinsic responsiveness of PMNs to hepoxilin A3 does not change with age.

We previously found that oral supplementation with α-Toc mitigates the harmful inflammatory response during pneumococcal infection of aged mice (Bou Ghanem et al., 2014). α-Toc treatment of PMNs from non-aged donors reduces transmigration across lung epithelial cells in response to *S. pneumoniae* infection (Bou Ghanem et al., 2014), likely by reducing the expression of ligands/receptors on PMNs, such as CD18/CD11b/CD55 and CD47 required for the migration process across the epithelial barrier (Hurley et al., 2008; Bou Ghanem et al., 2014). However, we did not previously test whether transmigration of PMNs from aged donors might also be diminished by α-Toc treatment. We found here that α-Toc treatment had a variable, donor-specific effect on PMN transmigration, consistent with previous research indicating diverse responses to oral α-Toc supplementation among individuals (Wu and Meydani, 2008; Belisle et al., 2010). In addition, α-Toc treatment decreased the average transepithelial migration of PMNs isolated from young and elderly donors to an equivalent degree. In contrast, vitamin E supplementation improves the function of other immune cells (CD4^+^ T-cells and macrophages) to a much greater extent in the elderly than the young (Adolfsson et al., 2001; Meydani et al., 2005; Marko et al., 2007, 2009).

Previous studies have revealed an age-associated decline in the ability of PMNs to kill several pathogens (Wenisch et al., 2000), including *S. pneumoniae* (Simell et al., 2011), after opsonization with whole serum or with the combination of complement and specific antibodies. Optimal bacterial killing by PMNs *ex vivo* requires complement (Esposito et al., 1990) and complement deficiency is associated with recurrent *S. pneumoniae* infections (Ram et al., 2010), indicating that complement plays an important role in host resistance to pneumococcus. To investigate age-dependent differences in complement-mediated bacterial killing by PMNs, we opsonized pneumococci with complement rather than homologous sera, avoiding the potentially confounding variable of anti-pneumococcal antibodies that may have been generated by previous pneumococcal infection (Ferreira et al., 2013). We found that PMNs from elderly donors are more efficient at killing complement-opsonized pneumococci than PMNs from young donors, indicating that not all PMN anti-microbial pathways decline with age. This is consistent with findings indicating that the levels of complement components and complement receptors CR1 and CR3 do not diminish with aging (Bellavia et al., 1999; Simell et al., 2011).

Polymorphonuclear leukocyte (PMN) serine proteases are required for efficient PMN-mediated pneumococcal killing (Standish and Weiser, 2009), and neutrophil elastase activity in the serum and bronchoalveolar lavage increases with age (Varga et al., 1992; Meyer et al., 1998; Paczek et al., 2009). Thus, consistent with our observation of enhanced killing of *S. pneumoniae* by PMNs from aged individuals, we found that although the amount of neutrophil elastase and cathepsin G of PMNs isolated from young or elderly donors were indistinguishable, their average activities were higher in PMNs from elderly donors. These results suggest that aging may be associated with changes in protease activity regulation, a process that is influenced by sequestration within the cell (Reeves et al., 2002; Pham, 2006) and inhibition by proteoglycans (Baici et al., 1980) or specific serine protease inhibitors, such as α1-anti-trypsin and secretory leukocyte protease inhibitor (SLP1; Pham, 2006). Age-driven changes in these inhibitors remain largely unexplored (Kida et al., 1992; Meyer et al., 1998). In addition to their role in killing pneumococci, serine proteases can exacerbate inflammation and augment lung damage (Sandhaus and Turino, 2013), indicating that age-driven increases in their activity could contribute to the exacerbated lung damage that accompanies bacterial pneumonia in the elderly.

A key finding of this study is that α-Toc treatment *ex vivo* boosted the serine protease activities of PMNs from young donors, as well as their ability to kill pneumococci. Since neutrophil elastase and cathepsin G are synthesized and packaged into azurophilic granules during early PMN development in the bone marrow (Gullberg et al., 1997; Pham, 2006; Cowland and Borregaard, 2016), our *ex vivo* α-Toc treatment of PMNs isolated from the blood is unlikely to trigger the production of serine proteases. Rather, the relatively short (1 h) exposure to α-Toc in this study likely induces a change in enzymatic activity regulation. Notably, whereas α-Toc enhanced the resistance of aged mice to pneumococcal lung infection, we found here that α-Toc treatment of PMNs from elderly human donors did not increase PMN protease or bacteriocidal activity. It is possible that, because the levels of bacteriocidal and protease activities in PMNs from elderly donors are higher than that of PMNs from younger donors, the dynamic range of these assays is too limited to detect α-Toc-mediated increases. In addition, given the potentially pleomorphic effects of weeks-long oral supplementation with α-Toc, this vitamin may boost some aspect of PMN function not tested in these *in vitro* assays. Regardless, these findings suggest that one mechanism by which α-Toc may boost host defense against *S. pneumoniae* is by enhancing the bacteriocidal serine protease activity of PMNs.

## Ethics statement

This study was carried out in accordance with the recommendations of “Investigation Review Board” with written informed consent from all subjects. All subjects gave written informed consent in accordance with the Declaration of Helsinki. The protocol was approved by the “Tufts Medical Center Human Investigation Review Board.”

## Author contributions

EB designed research, conducted research, analyzed data and wrote paper. JNL and BJ conducted research. SM designed research and provided essential reagents. AP and JML designed research and wrote paper and had primary responsibility for final content. All authors read and approved the final manuscript.

## Funding

This work is in part supported by the National Institute on Aging (NIA) and the American Federation for Aging Research (AFAR) (to AP), the A.S.P.E.N Rhoads Foundation (to EB) and U.S. Department of Agriculture Contract 58-1950-4-003 (to SM). EB is the recipient of the A.S.P.E.N 2013 and 2014 Abbott Nutrition grant. AP is the recipient of a 2012 Paul B. Beeson Career Development Award in Aging Research funded by the NIA (grant # 5K08AG042825) and AFAR.

### Conflict of interest statement

The authors declare that the research was conducted in the absence of any commercial or financial relationships that could be construed as a potential conflict of interest.
